# Amelioration of Diabetes and Painful Diabetic Neuropathy by *Punica granatum* L. Extract and Its Spray Dried Biopolymeric Dispersions

**DOI:** 10.1155/2014/180495

**Published:** 2014-05-27

**Authors:** K. Raafat, W. Samy

**Affiliations:** ^1^Department of Pharmaceutical Sciences, Faculty of Pharmacy, Beirut Arab University, Beirut 115020, Lebanon; ^2^Department of Pharmaceutical Technology, Faculty of Pharmacy, Beirut Arab University, Beirut 115020, Lebanon; ^3^Department of Industrial Pharmacy, Faculty of Pharmacy, Alexandria University, Alexandria 21521, Egypt

## Abstract

*Aims.* To evaluate the effect of *Punica granatum* (*Pg*) rind extract and its spray dried biopolymeric dispersions with casein (F1) or chitosan (F2) against *Diabetes mellitus* (DM) and diabetic neuropathy (DN). *Methods.* We measured the acute (6 h) and subacute (8 days) effect of various doses of *Pg*, F1, and F2 and the active compounds on alloxan-induced DM mouse model. We evaluated DN utilizing latency tests for longer period of time (8 weeks). In addition, the *in vivo* antioxidant activity was assessed utilizing serum catalase level. *Results.* The results proved that the highest dose levels of *Pg* extract, F1, F2 exerted remarkable hypoglycemic activity with 48, 52, and 40% drop in the mice glucose levels after 6 hours, respectively. The tested compounds also improved peripheral nerve function as observed from the latency tests. Bioguided fractionation suggested that gallic acid (GA) was *Pg* main active ingredient responsible for its actions. *Conclusion. Pg* extract, F1, F2, and GA could be considered as a new therapeutic potential for the amelioration of diabetic neuropathic pain and the observed *in vivo* antioxidant potential may be involved in its antinociceptive effect. It is highly significant to pay attention to *Pg* and GA for amelioration and control of DM and its complications.

## 1. Introduction

*Diabetes mellitus* (DM) is a major endocrine disorder and the global annual cost of treating DM and its complication could reach US $ trillion [1]. Amelioration of DM is a high priority in medical research. Self-management of DM is cornerstone to achieving good glycemic control and reducing the risk of developing microvascular (retinopathy, nephropathy, and neuropathy) and macrovascular (cardiovascular and cerebrovascular) complications [2]. Natural extracts have been used in management of diabetes and its related complications because they are safe and readily available [3]. Many plant species are used in folk medicine for their hypoglycemic properties and therefore potentially used for treatment of DM [4].

*Punica granatum* (*P. granatum*) L., universally known as pomegranate, from family Punicaceae, is a distinctive fruit bearing plant with various medicinal and dietary importance, native to the Middle East.* P. granatum* has shown to possess free radical scavenging, anticarcinogenic, anti-inflammatory, and effectiveness in the treatment of cancer, cardiovascular disease, Alzheimer's disease, arthritis, and erectile dysfunction [5]. Folk medicine suggests some possible benefits of* P. granatum* to diabetes and some related complications. Some* in vitro* studies were done to show the antihyperglycemic effect of* P. granatum* [6, 7]. The available research suggests that ellagic acid is the effective antihyperglycemic compound in* P. granatum *[8]. None of these investigations were done to show the antinociceptive effect of* P. granatum* on diabetic neuropathy (DN).

Oxidative stress, mediated mainly by hyperglycemia-induced generation of free radicals, helps in the development and progression of DM and its complications [4]. Diabetic peripheral neuropathy, which is one of the most frequent long-term complications of DM, is frequently accompanied with inferior quality of life [9]. This complication occurs in about one-quarter of diabetic patients [10]. Painful diabetic neuropathy is associated with symptoms and signs such as burning, tingling, or lancing type of spontaneous pain, allodynia, and hyperalgesia [11]. Thus, novel therapeutic targets are required for the satisfactory treatment of diabetic neuropathic pain [9, 12]. Abnormal free radicals high levels cause membrane damage leading to decline of antioxidant defense mechanisms causing cell and tissue damage [13]. The recent strategy for alleviating the oxidative damage in DM is based on supplementation with certain dietary antioxidants such as vitamins E and C and flavonoids [14].

Due to its unique physicochemical properties, casein, a natural biopolymeric surfactant, has a potential use in the preparation of conventional and novel pharmaceutical drug delivery systems. A number of* in vitro* and* in vivo* studies showed that casein is a suitable material for efficient drug delivery. Researchers have noted that casein has evolved to be easily degradable by the digestive enzymes proteases. Casein microspheres, either plain or cross-linked, could be promising parenteral biodegradable carriers for sustained delivery of drugs when administered via intramuscular, intraperitoneal, or direct intratumoral or intra-arterially for embolization in solid tumor deposits [15].

Chitosan, another biodegradable polymer, is a linear polysaccharide composed of randomly distributed (1–4)-linked d-glucosamine and N-acetyl-d-glucosamine [16]. Studies on the possible biomedical applications of chitosan have been adapted due to its low cytotoxicity, biodegradability, and antimicrobial activity [17–19].

Therefore, the aim of the present work involves the study of possible hypoglycemic,* in vivo *antioxidant, and diabetic neuropathy management effects of ethanolic rind extract of* P. granatum* and its biodegradable polymeric dispersions with either casein or chitosan.

## 2. Materials and Methods

### 2.1. Materials

Dried* P. granatum* L. fruit rind was purchased commercially from Ibn-Al-Nafess herbalist, Beirut, Lebanon. The solvents used in extraction and standards, alloxan, casein and chitosan (150 kDa) were purchased from Sigma-Aldrich (St. Louis, USA). Glibenclamide (GB) was commercially purchased (Lansa Pharm., China). Tramadol hydrochloride was obtained commercially (Deutsche Labs, Germany). All other chemicals were analytical grade chemicals.

### 2.2. Preparation of Plant Extract

The dried rind was extracted using 80% ethanol. The ethanolic extract was dried in a rotary evaporator (Buchi, Germany) at temperature 40°C under vacuum. The rind was authenticated with a reference sample, and a voucher specimen (PS-13-11) was deposited in the faculty herbarium.

### 2.3. Preparation of the Spray Dried Polymeric Dispersions

Casein was dissolved in 0.1 N NaOH in a concentration of 1.5% w/w. The partially dried extract (soft extract) of* P. granatum* was then added and mixed using a magnetic stirrer to have an extract/polymer ratio of 1 : 1. The polymeric dispersion was then neutralized with a few drops of 1 N HCl till a pH of 7.4 before feeding into LabPlant Mini-Spray dryer SD-06AG (LabPlant, UK). Inlet temperature was adjusted at 165°C and the outlet at 75–80°C. The dried product (F1) was collected and kept in a desiccator till further testing.

For chitosan dispersions (F2), chitosan solution (1.5% w/w) was prepared in 1% acetic acid and the soft extract was added to the solution to have an extract/polymer ratio of 1 : 1. The dispersion was then fed into the spray dryer as described above. The dried products were collected and kept in desiccators till further testing. Placebo dispersions of the polymers were prepared for control testing.

At the used extract/polymer ratios, the produced powders should have 1 mg* Pg* extract/2 mg of either of the dried formula (that the given* Pg* extract doses of 25, 50, and 100 mg/kg are equivalent to a powder dose; of either F1 or F2; of 50, 100, and 200 mg/kg, resp.).

### 2.4. HPLC Analysis of* P. granatum *Ethanolic Extract

Chromatographic optimization was done using the HPLC conditions and the investigation of columns with different packing materials, different wavelengths from 200 nm to 400 nm, and different mobile phase systems. An ideal chromatographic separation was achieved using RP-C18 endcapped Lichrospher column (250 × 4.6 mm I. D.; 5 *μ*M particle size) (Merck), at 40°C [20]. Isocratic elution was performed using (43 : 57) methanol/phosphate buffer 34.1 mM and pH 2.1. The flow rate was adjusted to 1 mL/min.

### 2.5. Determination of Total Phenolic Compounds in the Extract and Spray Dried Polymeric Dispersions

Generally, measurement of color occurred by reaction between Folin-Ciocalteu's phenol reagent [21, 22], and extract is a preferred method for the determination of the phenolic compounds, because the majority of their ingredients are polyphenols [21, 23]. Total contents of the phenolic compounds in the extracts were determined by Folin-Ciocalteu's method [22] as gallic acid equivalents (GAE) [23, 24]. In brief, 250 *μ*L of Folin-Ciocalteu's phenol reagent was mixed with 50 *μ*L of the samples, and 500 *μ*L of 20% water solution of Na_2_CO_3_ was added to the mixture. Mixtures were vortexed and completed with water to 5 mL. As control, reagent without addition of extract was used. After incubation of the samples at ambient temperature for 30 min, their absorbance was measured at 765 nm. The calibration curve was assembled by using fresh prepared gallic acid solutions as a base in calculations of total phenolic compound contents in the extracts. Experiments were repeated three times for every extract and the total phenolics were given in average values as GAE (mg gallic acid/g extract) [25, 26]. For the calibration curve, 10 mg of gallic acid was dissolved in 10 mL of MeOH utilizing an ultrasonic bath (stock solution). Various dilutions of stock solution were prepared and were determined by Folin-Ciocalteu's method [22, 27]. The experiments were repeated three times for every dilution and a calibration curve was created [24].

### 2.6. Content and* In Vitro* Release Pattern of the Dispersions

An amount equivalent to 8 mg of the soft extract was digested in 100 mL PBS pH 7.4. After sonication for 15 minutes, the content was assayed for the total phenolics by Folin-Ciocalteu's method [22] after proper dilution.

The* in vitro* release pattern of the extract was estimated by placing an amount equivalent to 8 mg of each formulation in a dialysis tubing (Visking Dialysis Tubing) closed by tubing closures at one end, 2 mL of the release medium was then added, and the other tube end was also closed by the tubing closure. The tube assembly was clamped to the paddle of a dissolution rate apparatus immersed in 500 mL PBS (pH 7.4) placed in the dissolution apparatus jar. The temperature was adjusted at 35°C, while the paddle was rotated at 50 rpm. Samples of 5 mL each were withdrawn at fixed time intervals and replaced with prewarmed buffer solution. Each sample was assayed by Folin-Ciocalteu's method and the average reading was calculated.

### 2.7. Bio-Guided Chromatographic Fractionation and Identification of the Effective Compounds

The* P. granatum* ethanolic extract (*Pg*) was fractionated using column chromatography. Preparative chromatography column, 50 mm diameter and 100 cm height, was used. Elution was done using ethyl acetate, formic acid, water, and hexane at ratios of 70 : 7.5 : 7.5 : 15, respectively, as mobile phase and silica gel as stationary phase. During the entire chromatography process the eluent was collected in a series of over 200 fractions by time.

Each fraction was tested in the same way as the test solutions in this study using* in vivo *alloxan-diabetic mice. The most active fraction was analyzed using RP-HPLC. The injection volume was 20 *μ*L and UV detection was performed using UV-detector at 270 nm, which has the highest absorbance for the tested active fraction, using JASCO spectrophotometer (JASCO), and confirmed utilizing TLC and standard steeping method utilizing standard calibration curves.

### 2.8. Animals

Male Swiss-Webster mice (Faculty of Pharmacy, Beirut Arab University) were accommodated for one week prior to the experimentation. The environment consisted of standard mouse cages with a 12 h light/dark cycle. The temperature was 22 ± 1°C; animals had open access to water and standard laboratory pellets (20% proteins, 5% fats, and 1% multivitamins) [26, 28]. The mice were kept in those conditions for a seven-day period of adaptation prior to the beginning of the experiment. Sixteen hours before the experiment, mice were fasted overnight but allowed open access to water. All animal care and experiments were done in accordance with Lebanese Ministry of Higher Education and with the approval of Beirut Arab University Institutional Review Board.

### 2.9. Diabetes Induction

Experimental DM was induced by intraperitoneal (IP) injection of freshly prepared alloxan dissolved in sterile saline (0.9%) every 48 h for three times at a dose of 180 mg/kg. Fasting glucose levels in the blood samples obtained from the tail of each mouse 72 h after the last alloxan injection were measured with Accu-Chek Active glucose strips in Accu-Chek Active Test Meter (Roche, USA). The glucose levels were expressed as mg/dL. Glucose (5%) was added to mice drinking water. The mice with blood glucose level more than 200 mg/dL were considered to be diabetic and were used in the experiment.

### 2.10. Acute Antidiabetic Effect of the* P. granatum* Extract in Alloxan-Induced Diabetic Mice

The diabetic mice were divided into sixteen groups of seven animals each. The animals of each group had a single IP injection as follows.

- Group I received only vehicle (0.9% sterile saline) and served as control.
- Group II received GB dissolved in DMSO as reference drug (5 mg/kg).
- Groups III, IV, and V received the* Pg* extract dissolved in vehicle at doses of 25, 50, and 100 mg/kg, respectively.
- Groups VI, VII, and VIII received F1, suspended in vehicle at doses equivalent to 25, 50, and 100 mg* Pg* extract/kg, respectively.
- Group VIII received placebo F1 suspended in vehicle (200 mg/kg).
- Groups IX, X, and XII received F2, suspended in vehicle at doses equivalent to 25, 50, and 100 mg* Pg* extract/kg, respectively.
- Group XIII received placebo F2 suspended in vehicle (200 mg/kg).
- Groups XIV, XV, and XVI received gallic acid (GA) dissolved in DMSO, at doses of 3, 6, and 12 mg/kg to the animals, respectively.

Blood samples were collected from the tail just prior to and at 0.5, 2, and 6 h after dosing. Blood glucose and body weight were determined.

### 2.11. Subacute Antidiabetic Effect of the* P. granatum* Extract in Alloxan-Induced Diabetic Mice

The action of* Pg *was also tested during a longer duration of treatment. The mice were divided into groups containing healthy and diabetic animals. Group I (healthy mice, *n* = 7) received only vehicle IP for 7 days and served as control [4]. The diabetic mice were divided into sixteen groups (II–XVII) of seven animals each. The animals of each group were treated for 7 days with a daily IP injection as follows.

- Group II received only vehicle (0.9% sterile saline) and served as diabetic control.
- Group III received GB dissolved in DMSO as reference drug (5 mg/kg, IP).
- Groups IV, V, and VI received* Pg *extract dissolved in vehicle at doses of 25, 50, and 100 mg/kg, respectively.
- Groups VII, VIII, and IX received F1 suspended in vehicle at doses equivalent to 25, 50, and 100 mg* Pg* extract/kg, respectively.
- Group X received placebo F1 suspended in vehicle 200 mg/kg.
- Groups XI, XII, and XIII received* Pg* F2 suspended in vehicle at doses equivalent to 25, 50, and 100 mg* Pg* extract/kg, respectively.
- Group XIV received placebo F2 suspended in vehicle 200 mg/kg.
- Groups XV, XVI, and XVII received GA, dissolved in DMSO, at doses of 3, 6, and 12 mg/kg, respectively.

The blood glucose level of each animal was monitored on the 1st, 3rd, 5th, and 8th days after 6 h of each injection given every other day.

The antioxidant enzyme (catalase) levels were measured and the body weights of the animals were recorded at the same day.

### 2.12. Management of Diabetic Neuropathy

After six weeks of induction of diabetes in animals, DN success rate (i.e., loss of sensory of thermal sensitivity significantly below 10 s [29]) was ca. 85%, and their neurological function was tested at one-week intervals for eight weeks, with tramadol (TRA) 10 mg/kg as a positive control, using the following tests.

#### 2.12.1. Hot Plate Latency Test

The animals were positioned one at a time on the hot plate (hot plate analgesia meter, Ugo Basile, Italy) that is maintained at a temperature of 55 ± 0.1°C. Response latency either to jump or to a hindpaw lick was monitored by means of an electronic timer. To avoid tissue damage, a cut-off time of 30 s was selected [30].

#### 2.12.2. Tail Flick Latency Test

In brief, a beam of light was focused on the dorsal surface of the mouse tail using tail flick apparatus with appropriate tail flick mice restrainer (Hugo Sachs Elektronik, Germany) and the time until the tail flicked was measured. The tail withdrawal latency, time from onset of the radiant heat to the withdrawal of the tail, was recorded with a timer. The light intensity in the apparatus was set so that the baseline tail withdrawal latency was about 5.6 s in all mice. A cut-off time of 10.00 ± 0.50 s was set with the purpose of preventing tissue damage [30].

### 2.13. Estimation of Antioxidant Activity

Catalase (CAT) activity was determined in serum using the modified method described before in the literature [31]. CAT activity was expressed as kU/L.

### 2.14. Statistical Analysis

All values are presented as means ± SEM. Statistical differences between the test and the control were tested by one-way analysis of variance (ANOVA) followed by the Student-Newman-Keuls test using the “OriginPro” statistic computer program. A difference in the mean values of *P* ≤ 0.05 was considered to be statistically significant.

## 3. Results 

### 3.1. HPLC Analysis of* Pg* Extract

After the bioguided fractionation, the most active* P. granatum* extract fraction was injected in the HPLC instrument to study its pattern. The most active fraction major peak was GA (11.4%). Ellagic acid was not detected in the active fraction. The concentration of GA was measured using the area under peak utilizing GA calibration curve.

### 3.2. Total Phenolic Compounds Using Folin-Ciocalteu Assay

Total phenolic content of 0.15 ± 0.01 mg gallic acid/g* Pg* extract, using Folin-Ciocalteu Reagent (FCR), was found in* Pg *extract. All absorbance was measured at 765 nm. Results are mean ± SEM of three parallel measurements, and *P* < 0.01, when compared to the control.

### 3.3. Content and* In Vitro *Release Pattern

**Figure 1:**
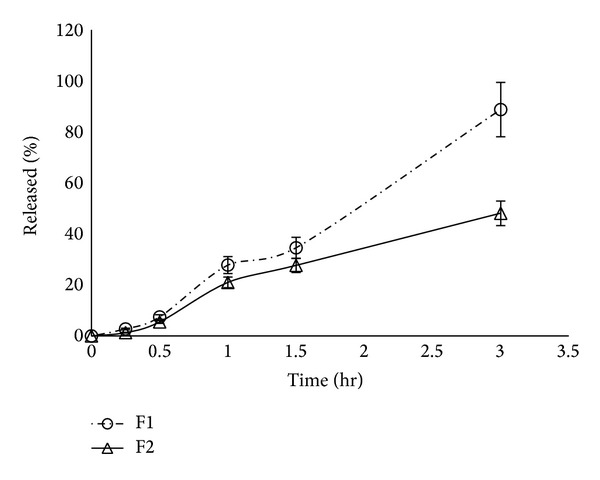
*In vitro* release pattern of* P. granatum* L. extract from different biopolymeric dispersions of casein (F1) and chitosan (F2).

All the prepared powders contained 98 ± 2% of the claimed extract content calculated as mg gallic acid/g extract. The* in vitro* release pattern of the tested powders showed that F1 had a faster release pattern with 7.5% released after 30 minutes compared with 5.5% in case of F2. The difference in release was highly observed after 3 hours with F1 releasing more than 85% of the extract compared with less than 50% for F2. Retardation of drug release from such dispersions of casein is commonly achieved via surface cross-linkage (Figure 1).

### 3.4. Acute Antidiabetic Effect of the* Pg *Extract in Alloxan-Induced Diabetic Mice

The acute antidiabetic effect of various doses of the* Pg *extract in diabetic animals is summarized in Table 1. The* Pg *extract at all doses (25, 50, and 100 mg/kg) showed a significant effect compared to the control, with blood glucose levels dropping after 6 h of administration to 36.4, 34.2, and 48.4%, respectively. GB, the positive control, prevented the drastic increase of blood glucose after 1 h of glucose loading. On the other hand, GB reduced the glucose level, 2 and 6 h after the glucose loading. Dispersion F1 at all doses (equivalent to 25, 50, and 100 mg* Pg *extract/kg) showed significant decrease in blood glucose level after 6 h dropping to 40.6, 40.0, and 52.4%, respectively. Similar to F1, F2 showed significant decrease in blood glucose level after 6 h at all doses (equivalent to 25, 50, and 100 mg* Pg *extract/kg) with the glucose level dropping to 30.2, 42.6, and 40.6%, respectively. On the other hand, placebos F1 and F2 did not significantly reduce the glucose level 6 h after the glucose loading. In case of GA, standard GA at the lowest dose (3 mg/kg) did not show a significant effect from that of control after 6 h of glucose administration. At higher doses (6 and 12 mg/kg) GA showed a significant effect, with blood glucose levels dropping to 36.1 and 36.4%, respectively, compared to that of control after 6 h of glucose administration.

### 3.5. Subacute Effect of the* Pg *Extract in Alloxan-Induced Diabetic Mice

As shown in Table 2, the blood glucose levels of diabetic control (positive control) mice were significantly higher than those of the control (negative control) mice during the experiment period. The highest reduction in blood glucose using* Pg* extract was observed with a dose of 100 mg/kg, showing 54.2% reduction in blood glucose levels on the 8th day compared with 17.7 and 6.4% reduction in case of 25, 50 mg/kg doses, respectively.

The F1 at all doses (equivalent to 25, 50, and 100 mg* Pg *extract/kg) showed significant decrease in blood glucose level on the 8th day compared to that of the diabetic control with the mice glucose levels dropping to 40.2, 40.9, and 50.5%, respectively. Similarly, F2 at all doses (equivalent to 25, 50, and 100 mg* Pg *extract/kg) showed significant decrease in blood glucose level on the 8th day dropping to 43.7, 44.1, and 49.0%, respectively. Placebos F1 and F2 did not significantly reduce the glucose level on the 8th day compared to that of the diabetic control.

For GA, standard GA showed a significant effect compared to that of diabetic control on the 8th day at all doses (3, 6, and 12 mg/kg), with blood glucose levels dropping to 51.6, 53.3, and 43.7%, respectively.

During the subacute administration, mice treated with GB and various doses of the* Pg *extract, F1, F2, and GA were also monitored for changes in weight (Table 3). The* Pg *extract showed 5.9, 30.7, and 21.9% increase in body weight at doses of 25, 50, and 100 mg/kg, respectively, on the 8th day. In case of F1 at doses equivalent to 25, 50, and 100 mg* Pg *extract/kg showed 9.8, 10.5, and 17.8% increase in body weight on the 8th day, respectively. Similarly, F2 at doses equivalent to 25, 50, and 100 mg* Pg *extract/kg showed 9.8, 10.5, and 17.8% increase in body weight on the 8th day. Placebos F1 and F2 did not significantly show any increase in body weight on the 8th day compared to that of the diabetic control. Also GA at all doses (3, 6, and 12 mg/kg) showed a significant increase in body weight on the 8th day, of 23.9, 18.9, and 6.5%, respectively.

In order to evaluate* in vivo *antioxidant effect of the tested* Pg* extract and its polymeric dispersions, CAT level in serum of each mouse was monitored on 1st, 3rd, 5th, and 8th days after administration. As shown in Table 4, diabetic mice were monitored for changes in serum CAT level after treatment with GB and various doses of the* Pg *extract, F1, F2, and GA. The* Pg *extract at doses of 25, 50, and 100 mg/kg had a gradual rise in serum CAT activity to reach a significant difference on 5th (1.0, 9.2, and 15.1%, resp.) and 8th day (4.1, 6.1, and 15.5%, resp.) as compared with diabetic control mice. Polymeric dispersion F1 at doses equivalent to 25, 50, and 100 mg* Pg *extract/kg had a gradual rise in serum CAT activity to reach significant differences on 5th (2.7, 8.7, and 14.5%, resp.) and 8th day (5.2, 6.1, and 19.8%, resp.) as compared with diabetic control mice. Similarly, F2 had a gradual rise in serum CAT activity to reach a significant difference on the 8th day with CAT activities of 3.3, 4.0, and 10.9% for doses equivalent to 25, 50, and 100 mg* Pg *extract/kg, respectively. Placebos F1 and F2 did not significantly show any increase in serum CAT level compared to that of the diabetic control. GA at doses 6 and 12 mg/kg had also shown a gradual rise in serum CAT activity to reach a significant difference on the 8th day (3.4 and 3.1%, resp.) as compared with diabetic control mice.

The acute and subacute antihyperglycemic activity of the* Pg *extract, F1, F2, and GA were shown to be more potent and prolonged than those of GB.

### 3.6. Management of Diabetic Neuropathy

Deterioration of peripheral nerve conduction is a milestone indicator for diabetic patients having peripheral neuropathy [32–34]. So, we examined the effect of* Pg *extract, F1, F2, and GA treatment on sensory function by measuring the thermal latency with tail flick and hot plate tests on the 8th week after alloxan injection.

**Figure 2:**
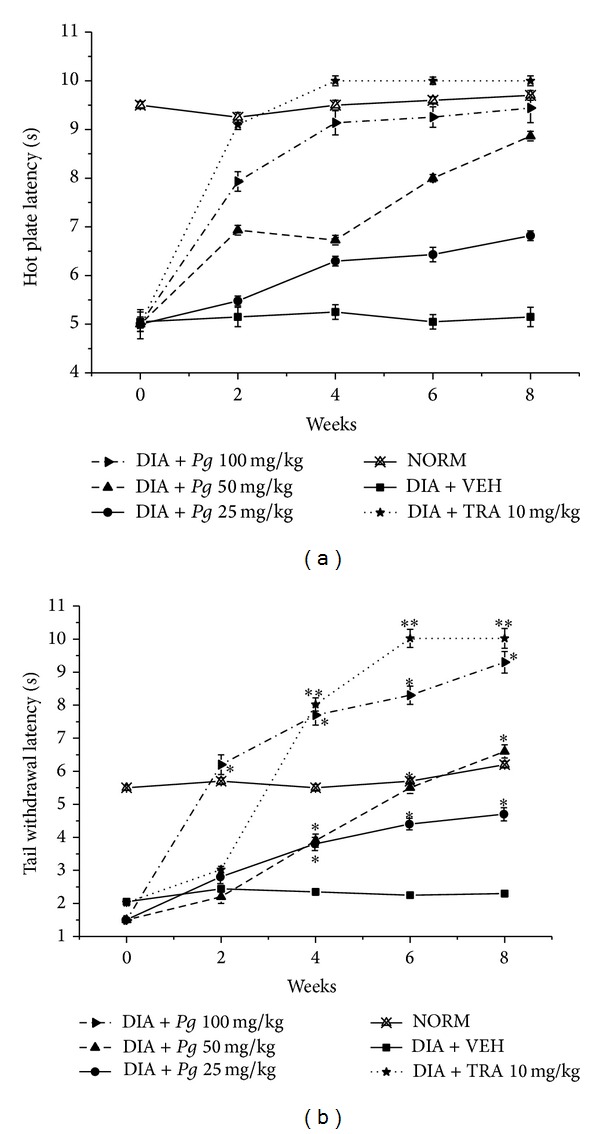
Effect of* P. granatum *ethanolic extract (*Pg*) and tramadol (TRA) 10 mg/kg, as positive control, on the hot plate and tail withdrawal latencies in alloxan-induced diabetic mice. (a) Hot plate latency: NORM: normal control mice (crossed-triangles, straight line); DIA + VEH: diabetic animals treated with vehicle as control (closed-squares, straight-line); positive control TRA 10 mg/kg: alloxan treated mice with TRA 10 mg/kg (solid-stars, dotted-line); DIA +* Pg* 25 mg/kg: diabetic animals treated with* Pg* 25 mg/kg (solid-circles, straight-line); DIA +* Pg* 50 mg/kg: diabetic animals treated with* Pg* 50 mg/kg (up-triangles, dashed-line); DIA +* Pg* 100 mg/kg: diabetic animals treated with* Pg* 100 mg/kg (right-triangles, dashed-dotted-line). (b) Tail withdrawal latency: NORM: normal control mice (crossed-triangles, straight line); DIA + VEH: diabetic animals treated with vehicle as control (closed-squares, straight-line); positive control TRA 10 mg/kg: alloxan treated mice with TRA 10 mg/kg (solid-stars, dotted-line); DIA +* Pg* 25 mg/kg: diabetic animals treated with* Pg* 25 mg/kg (solid-circles, straight-line); DIA +* Pg* 50 mg/kg: diabetic animals treated with* Pg* 50 mg/kg (up-triangles, dashed-line); DIA +* Pg* 100 mg/kg: diabetic animals treated with* Pg* 100 mg/kg (right-triangles, dashed-dotted-line). Data are expressed in mean ± SEM. “∗” means *P* < 0.05 compared with vehicle. “∗∗” means *P* < 0.01 compared with vehicle.

Treatment of the alloxan-induced diabetic mice with* Pg *extract markedly improved the thermal latency compared with TRA 10 mg/kg positive control (Figure 2(a)). Diabetic mice exhibited transient hyperalgesic response in thermal tests. On the 8th week after alloxan injection, treatment with* Pg *extract showed a marked improvement in hot plate latency compared to vehicle treated group by 33.3, 73.5, and 85.1% in doses of 25, 50, and 100 mg/kg, respectively (Figure 2(a)).

Nevertheless, treatment with all doses of* Pg *extract demonstrated a marked improvement in tail flick latency by ca. one-, two-, and threefold fordoses of 25, 50, and 100 mg/kg, respectively, compared to vehicle treated group (Figure 2(b)).

**Figure 3:**
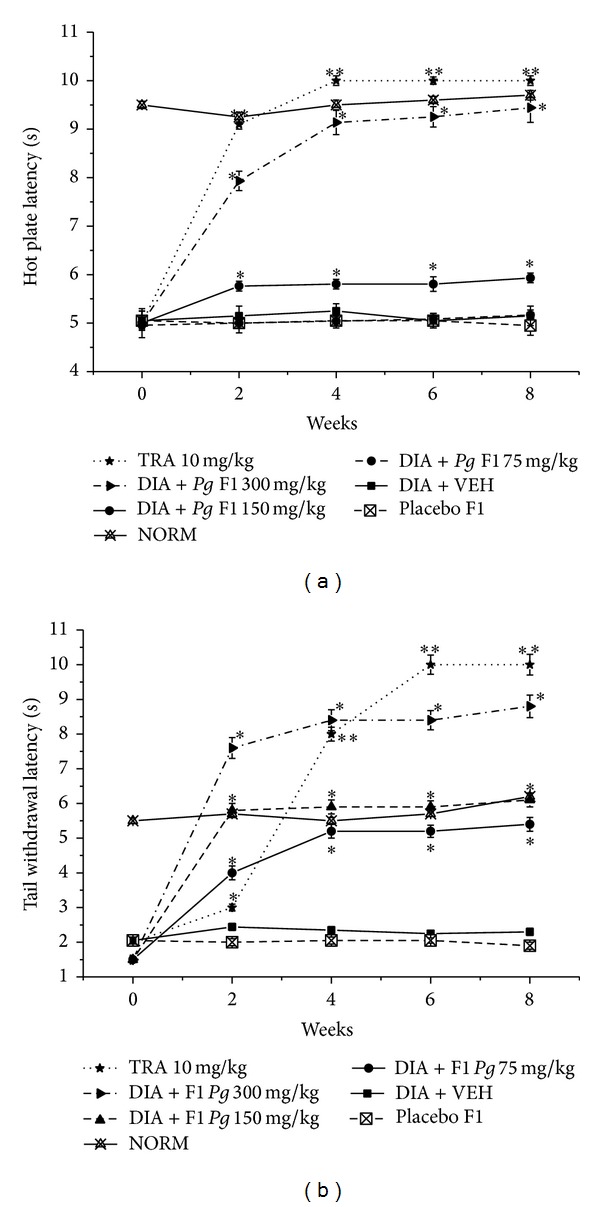
Effect of F1 and TRA 10 mg/kg, as positive control, on the hot plate and tail withdrawal latencies in alloxan-induced diabetic mice. (a) Hot plate latency: NORM: normal control mice (crossed-triangles, straight line); DIA + VEH: diabetic animals treated with vehicle as control (closed-squares, straight-line); placebo F1: diabetic animals treated with placebo F1 200 mg/kg (crossed-squares, dashed-line); positive control TRA 10 mg/kg: alloxan treated mice with TRA 10 mg/kg (solid-stars, dotted-line); DIA + F1 50 mg/kg: diabetic animals treated with F1 50 mg/kg (solid-circles, straight-line); DIA + F1 100 mg/kg: diabetic animals treated with F1 100 mg/kg (up-triangles, dashed-line); DIA + F1 200 mg/kg: diabetic animals treated with* Pg* F1 200 mg/kg (right-triangles, dashed-dotted-line). (b) Tail withdrawal latency: NORM: normal control mice (crossed-triangles, straight line); DIA + VEH: diabetic animals treated with vehicle as control (closed-squares, straight-line); placebo F1: diabetic animals treated with placebo F1 200 mg/kg (crossed-squares, dashed-line); positive control TRA 10 mg/kg: alloxan treated mice with TRA 10 mg/kg (solid-stars, dotted-line); DIA + F1 50 mg/kg: diabetic animals treated with F1 50 mg/kg (solid-circles, straight-line); DIA + F1 100 mg/kg: diabetic animals treated with F1 100 mg/kg (up-triangles, dashed-line); DIA + F1 200 mg/kg: diabetic animals treated with F1 200 mg/kg (right-triangles, dashed-dotted-line). Data are expressed in mean ± SEM. “∗” means *P* < 0.05 compared with vehicle. “∗∗” means *P* < 0.01 compared with vehicle.

On the 8th week, treatment with the lowest dose of F1 did not significantly improve hot plate latency, while higher doses (equivalent to 50 and 100 mg* Pg* extract/kg) of F1 markedly improved hot plate latency by 15.7 and 84.3%, respectively, compared to vehicle treated group (Figure 3(a)). Nonetheless, treatment with all doses of F1 the tail flick latency have markedly improved by ca. 1.5-, 1.7-, and 3-fold in F1 doses equivalent to 25, 50, and 100 mg* Pg* extract/kg, respectively (Figure 3(b)).

**Figure 4:**
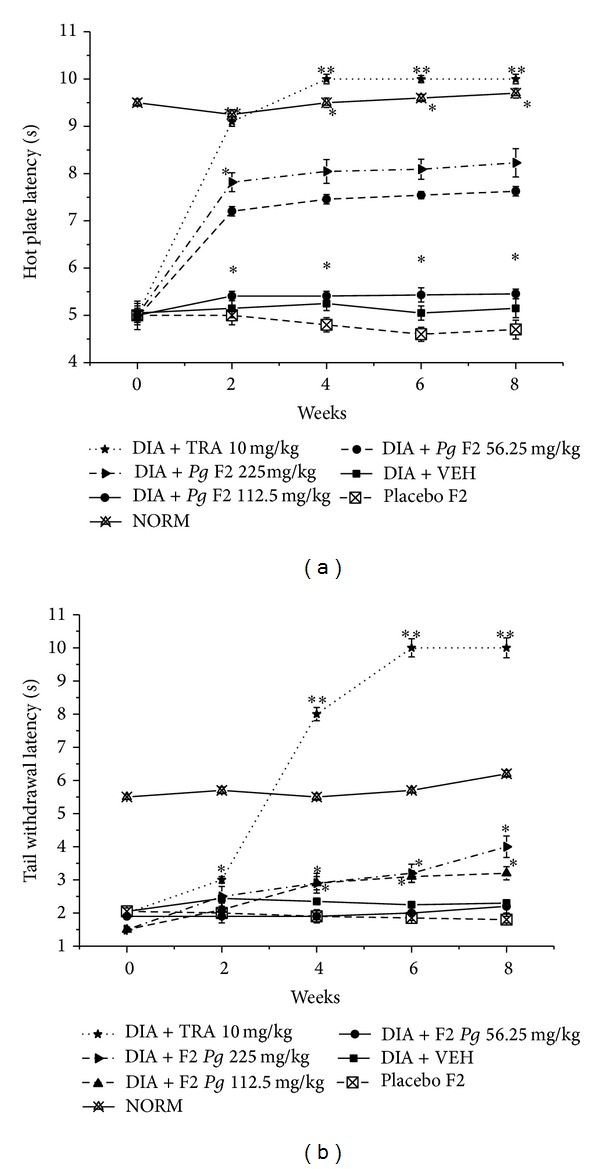
Effect of F2 and TRA 10 mg/kg, as positive control, on the hot plate and tail withdrawal latencies in alloxan-induced diabetic mice. (a) Hot plate latency: NORM: normal control mice (crossed-triangles, straight line); DIA + VEH: diabetic animals treated with vehicle as control (closed-squares, straight-line); placebo F2: diabetic animals treated with placebo F2 200 mg/kg (crossed-squares, dashed-line); positive control TRA 10 mg/kg: alloxan treated mice with TRA 10 mg/kg (solid-stars, dotted-line); DIA + F2 50 mg/kg: diabetic animals treated with F2 50 mg/kg (solid-circles, straight-line); DIA + F2 100 mg/kg: diabetic animals treated with F2 100 mg/kg (up-triangles, dashed-line). DIA +* Pg* 200 mg/kg: diabetic animals treated with F2 200 mg/kg (right-triangles, dashed-dotted-line). (b) Tail withdrawal latency: NORM: normal control mice (crossed-triangles, straight line); DIA + VEH: diabetic animals treated with vehicle as control (closed-squares, straight-line); placebo F2: diabetic animals treated with placebo F2 200 mg/kg (crossed-squares, dashed-line); positive control TRA 10 mg/kg: alloxan treated mice with TRA 10 mg/kg (solid-stars, dotted-line). DIA + F2 50 mg/kg: diabetic animals treated with F2 50 mg/kg (solid-circles, straight-line); DIA + F2 100 mg/kg: diabetic animals treated with F2 100 mg/kg (up-triangles, dashed-line). DIA + F2 200 mg/kg: diabetic animals treated with F2 200 mg/kg (right-triangles, dashed-dotted-line). Data are expressed in mean ± SEM. “∗” means *P* < 0.05 compared with vehicle. “∗∗” means *P* < 0.01 compared with vehicle.

Moreover, treatment with the lowest dose of F2 did not significantly improve hot plate or tail flick latencies, while higher doses (equivalent to 50 and 100 mg* Pg* extract/kg) of F2 markedly improved hot plate latency by 45.5 and 60.8%, respectively, and tail flick latency by ca. 0.3- and 0.9-fold compared to vehicle treated group (Figures 4(a) and 4(b)).

Both Placebos F1 and F2 did not significantly improve neither hot plate nor tail flick latencies (Figures 3 and 4).

**Figure 5:**
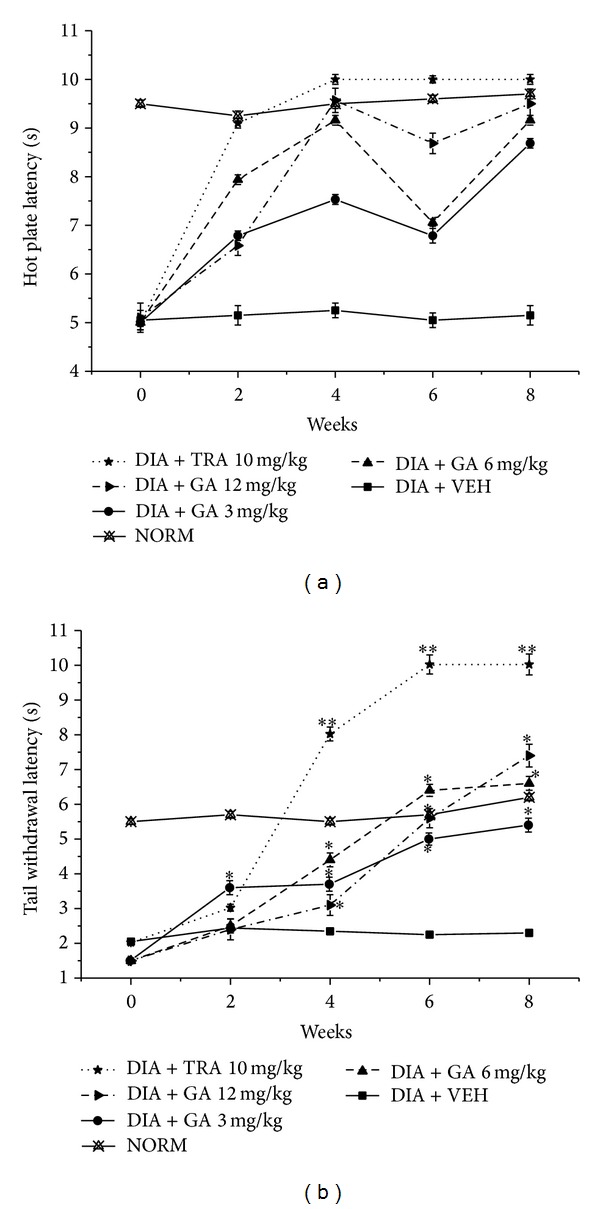
Effect of gallic acid (GA) and tramadol (TRA) 10 mg/kg, as positive control, on the hot plate and tail withdrawal latencies in alloxan-induced diabetic mice. (a) Hot plate latency: NORM: normal control mice (crossed-triangles, straight line); DIA + VEH: diabetic animals treated with vehicle as control (closed-squares, straight-line); positive control TRA 10 mg/kg: alloxan treated mice with TRA 10 mg/kg (solid-stars, dotted-line); DIA + GA 3 mg/kg: diabetic animals treated with GA 3 mg/kg (solid-circles, straight-line); DIA + GA 6 mg/kg: diabetic animals treated with GA 6 mg/kg (up-triangles, dashed-line). DIA +* Pg* 12 mg/kg: diabetic animals treated with GA 12 mg/kg (right-triangles, dashed-dotted-line). (b) Tail withdrawal latency: NORM: normal control mice (crossed-triangles, straight line); DIA + VEH: diabetic animals treated with vehicle as control (closed-squares, straight-line); positive control TRA 10 mg/kg: alloxan treated mice with TRA 10 mg/kg (solid-stars, dotted-line); DIA + GA 3 mg/kg: diabetic animals treated with GA 3 mg/kg (solid-circles, straight-line); DIA + GA 6 mg/kg: diabetic animals treated with GA 6 mg/kg (up-triangles, dashed-line); DIA + GA 12 mg/kg: diabetic animals treated with GA 12 mg/kg (right-triangles, dashed-dotted-line). Data are expressed in mean ± SEM. “∗” means *P* < 0.05 compared with vehicle. “∗∗” means *P* < 0.01 compared with vehicle.

In addition, treatment with GA (3, 6, and 12 mg/kg) showed a marked improvement in hot plate latency by 70.6, 78.6, and 86.3% and tail flick latency by ca. 1.5-, 2-, and 2.4-fold, respectively, compared to vehicle treated group (Figures 5(a) and 5(b)).

## 4. Discussion

Extended exposure to hyperglycemia promotes the development of microvascular and macrovascular complications associated with DM [35]. The high oxidative stress in diabetics considerably contributes to the complications of this disease [36] and excessive production of free radicals is a discovered phenomenon associated with diabetic complications [37]. Management of DM and diabetic complications with minimal side effect is still a major challenge to the medical system [35].

This directed scientists to broader exploration of potent natural antidiabetics with fewer side effects. In this study we chose* Pg *extract based on its folkloric use in treatment of DM in Lebanon. The current research was focused on assessing its possible antidiabetic and antinociceptive activities of* Pg* extract and its spray dried biodegradable polymeric dispersions, along with its most effective compound, GA.

There are a number of scientific reports proposing the antidiabetic potential of* Pg* extracts [5, 7, 8], whereas the active compound responsible for this action was assumed to be ellagic acid [8]. In the present work, bioguided fractionation utilizing column chromatography, TLC and RP-HPLC, indicated that gallic acid (GA) is the most effective compound.

The hypoglycemic action of the extract was observed to be dose dependent in hyperglycemic mice, with significant decrease of blood glucose level at the highest dose levels of 100 mg extract/kg.

It has been found that the highest dose levels of* Pg* extract (100 mg/kg), F1, F2, and GA (6 mg/kg) are the most effective doses in the acute and subacute groups. These doses have more significant effect on blood glucose level compared to that of the synthetic drug, GB. An initial increase in blood glucose levels after 0.5 and sometimes 2 hrs after treatment with* Pg* extract and GA was observed during testing the acute antidiabetic effect. This temporary initial hyperglycemia may be due to preglucose loading, while, at the same time, the test compounds did not start to give their effect directly after administration.

In the acute antidiabetic effect, F1 had shown a relatively higher reduction in glucose level compared with F2. This could be attributed to the micellar arrangement of casein that promotes solubilization within the release medium [15], thus improving the release. The same release pattern was seen in the* in vitro* release experiment with F1 showing faster release than F2.

In the subacute antidiabetic effect, due to F2 relatively slower release seen in its* in vitro* release pattern, F2 showed a relatively higher subacute effect compared to F1, an effect that could be beneficial for designing a long acting form of the extract for controlling DM.

Currently, much attention has been given on the role of oxidative stress. It has been suggested that oxidative stress may comprise the key and common events in the pathogenesis of different diabetic complications [38].

*Pg* extract, F1, F2, and GA showed a significant increase in body weight, as an evidence of alleviating of hyperglycemia, as demonstrated before with pharmacotherapies used in management of DM [3, 39].

It was suggested that the mechanism of action of GA appears to be through the regeneration of the damaged Langerhans *β*-cells and the potentiation of insulin release from the existing ones [40].

In our study, the activity of CAT decreased in diabetic mice as reported earlier [38, 41] which could be due to inactivation caused by alloxan-generated reactive oxygen species (ROS). Long-term treatment of DM with all doses, especially with the highest dose of the* Pg *extract, F1, F2, and GA, could have reversed the activities of this enzymatic antioxidant, which might be due to lessened oxidative stress as evidenced by the elevation in CAT activity.

*Pg* extract has long been known as having antioxidant molecules of gallic and ellagic acids [42, 43]. The most effective active fraction of our extract did not contain ellagic acid; on the other hand, it has shown a comparatively high percentage of GA. The effect of GA in the same concentration, relative to that present in the* Pg* extract, is comparatively showing equipotent antihyperglycemic and antioxidant activity to* Pg* extract, which may indicate that GA is the most effective bioactive component in* Pg* extract.

Administration of* Pg *extract, F1, F2, and GA also alleviated hyperalgesia in pain conditions compared to that of the positive control, tramadol (TRA).

These findings supply health care providers with a promising medication intended for symptomatic management of diabetic neuropathy, a safe antidiabetic agent, and a safe treatment of micro- and macrovascular complications of DM. Slow release dispersions (F2) may be more beneficial in long-term management (subacute antidiabetic effect) while rapid ones (F1) may be better for acute management.

In conclusion, the present study indicated that* Pg *extract as well as its biodegradable polymeric dispersions with casein (F1) and chitosan (F2) exerted remarkable hypoglycemic activity and improved peripheral nerve function, which might be due to GA, that prevents oxidative stress in diabetic animals beside its insulin-secretagogue action. Therefore, the observed* in vivo* antioxidant potential of the* Pg* extract might possibly be added to the mechanism of action responsible for its antinociceptive effect.

## Figures and Tables

**Table 1:** Acute effect of *P. granatum *ethanolic extract (*Pg*), F1, F2, and gallic acid (GA) on blood glucose of alloxan-induced diabetic mice (*n* = 7).

Group	Dose (mg/kg)	Mean blood glucose concentration ± SEM (mg/dL)			
		0 hr	0.5 hr	2 hr	6 hr
Diabetic control	—	202.79 ± 5.60	211.93 ± 4.50	214.21 ± 9.70	210.15 ± 7.30
GB	5	219.70 ± 3.70	223.64 ± 1.80	159.74 ± 2.10	130.64 ± 2.40**
*Pg *	25	218.29 ± 1.50	142.44 ± 2.80	181.56 ± 2.02	138.89 ± 1.45*
*Pg *	50	200.43 ± 2.10	240.93 ± 2.50	158.41 ± 2.60	131.96 ± 1.90*
*Pg *	100	249.92 ± 1.80	292.44 ± 2.80	228.56 ± 2.02	128.98± 1.54*
Placebo F1	200	208.10 ± 3.10	227.23 ± 2.10	222.62 ± 2.30	209.98 ± 2.90
F1	50	243.12 ± 1.30	158.44 ± 1.90	167.65 ± 2.10	144.33 ± 2.00*
F1	100	201.33 ± 1.70	130.44 ± 2.10	160.89 ± 2.90	120.77 ± 2.10*
F1	200	263.11 ± 2.90	139.56 ± 2.40	117.79 ± 2.80	125.33 ± 2.30*
Placebo F2	200	213.56 ± 3.10	202.17 ± 2.60	201.13 ± 2.80	229.56 ± 3.10
F2	50	211.55 ± 2.90	205.12 ± 1.30	146.82 ± 1.40	147.725 ± 2.80*
F2	100	214.65 ± 1.30	213.99 ± 1.90	186.83 ± 1.10	123.27 ± 1.20*
F2	200	240.44 ± 2.50	190.825 ± 1.90	172.165 ± 1.80	142.87 ± 2.20*
GA	3	201.29 ± 5.60	210.43 ± 4.50	213.71 ± 9.70	209.05 ± 7.30
GA	6	202.20 ± 3.70	222.14 ± 1.80	158.24 ± 2.10	129.14 ± 2.40**
GA	12	218.29 ± 1.50	142.44 ± 2.80	181.56 ± 2.02	138.89 ± 1.45*

SEM: standard error of the mean.

*P < 0.05 significant from the control animals.

**P < 0.01 significant from the control animals.

**Table 2:** Subacute effect of *Pg*, F1, F2, and GA on blood glucose of alloxan-induced diabetic mice (*n* = 7).

Group	Dose (mg/kg)	Mean blood glucose concentration ± SEM (mg/dL)			
		1st day	3rd day	5th day	8th day
Control	—	107.90 ± 2.50	109.80 ± 3.60	108.86 ± 3.20	117.50 ± 4.70
Diabetic control^a^	—	202.79 ± 5.60***	211.93 ± 4.50***	214.91 ± 9.70***	210.55 ± 7.30***
GB^b^	5	186.70 ± 3.70	179.53 ± 2.90	161.54 ± 2.40**	171.97 ± 3.10
*Pg* ^ b^	25	138.67 ± 2.60	87.54 ± 1.70	158.63 ± 2.30	135.50 ± 2.10**
*Pg* ^ b^	50	143.34 ± 1.90	119.67 ± 1.70	119.33 ± 1.90	119.10 ± 1.10**
*Pg* ^ b^	100	132.12 ± 1.30	112.98 ± 2.50	124.36 ± 2.60	111.50 ± 2.50*
Placebo F1	200	193.10 ± 3.10	212.66 ± 2.90	229.54 ± 2.80	226.33 ± 2.10
F1^b^	50	144.11 ± 1.20	129.40 ± 1.60	130.66 ± 2.40	125.88 ± 3.10*
F1^b^	100	120.33 ± 1.70	115.60 ± 2.40	128.65 ± 2.90	124.33 ± 3.20*
F1^b^	200	125.21 ± 2.90	115.50 ± 3.10	108.40 ± 2.40	104.32 ± 1.90*
Placebo F2	200	229.33 ± 2.50	226.89 ± 2.90	204.98 ± 3.10	265.44 ± 2.90
F2^b^	50	175.50 ± 2.50	119.90 ± 2.10	119.10 ± 1.30	118.50 ± 1.90*
F2^b^	100	118.65 ± 1.30	128.10 ± 1.30	122.49 ± 2.40	117.65 ± 2.10*
F2^b^	200	191.33 ± 3.90	94.54 ± 1.40	108.55 ± 3.40	107.43 ± 2.50*
GA^b^	3	198.10 ± 1.90	208.00 ± 2.20	111.36 ± 3.30	101.88 ± 2.22*
GA^b^	6	179.65 ± 3.10	134.66 ± 2.60	130.54 ± 2.90	98.23 ± 2.87*
GA^b^	12	168.33 ± 2.90	140.50 ± 3.50	137.25 ± 2.70	118.55 ± 3.50*

SEM: standard error of the mean.

*P < 0.05 significant from the control animals.

**P < 0.01 significant from the control animals.

***P < 0.001 significant from the control animals.

^a^Compared to vehicle control.

^b^Compared to diabetic control.

**Table 3:** Subacute effect of *Pg*, F1, F2, and GA on body weights in alloxan-induced diabetic mice (*n* = 7).

Group	Dose (mg/kg)	Mean body weight ± SEM (gm)			
		1st day	3rd day	5th day	8th day
Control	—	25.90 ± 0.50	26.00 ± 0.60	26.01 ± 0.97	26.62 ± 0.70
Diabetic control^a^	—	28.68 ± 0.70	27.10 ± 0.20	27.15 ± 0.80	27.69 ± 0.50
GB^b^	5	22.90 ± 0.70	28.17 ± 1.70	28.54 ± 0.40	30.37 ± 1.10*
*Pg* ^ b^	25	24.11 ± 2.60	27.70 ± 1.50	30.20 ± 1.60	30.40 ± 2.10**
*Pg* ^ b^	50	29.30 ± 1.90	29.20 ± 1.60	29.30 ± 1.80	31.50 ± 2.20**
*Pg* ^ b^	100	30.24 ± 2.60	30.40 ± 1.70	31.54 ± 3.90	31.98 ± 2.90*
Placebo F1	200	27.50 ± 2.10	27.50 ± 2.20	27.00 ± 2.40	26.88 ± 2.50
F1^b^	50	31.11 ± 1.20	31.00 ± 1.50	31.10 ± 1.60	31.50 ± 1.90
F1^b^	100	30.10 ± 1.40	29.50 ± 2.50	30.50 ± 2.40	31.70 ± 2.50*
F1^b^	200	31.00 ± 1.90	29.00 ± 3.50	32.60 ± 2.90	33.80 ± 2.80*
Placebo F2	200	24.50 ± 2.50	24.45 ± 2.60	24.01 ± 2.50	23.60 ± 2.40
F2^b^	50	27.30 ± 1.90	27.40 ± 1.80	27.5 ± 1.90	27.8 ± 2.00
F2^b^	100	28.40 ± 1.40	28.5 ± 1.50	28.90 ± 1.60	30.25 ± 2.50*
F2^b^	200	31.90 ± 1.90	32.00 ± 1.80	32.50 ± 1.90	33.11 ± 2.10*
GA^b^	3	29.20 ± 1.20	30.30 ± 1.30	30.50 ± 1.10	32.05 ± 1.20*
GA^b^	6	31.56 ± 1.10	32.67 ± 0.60	33.45 ± 1.00	34.10 ± 1.60*
GA^b^	12	30.20 ± 1.20	30.30 ± 1.30	29.50 ± 1.50	30.56 ± 1.40

SEM: standard error of the mean.

*P < 0.05 significant from the control animals.

^a^Compared to vehicle control.

^b^Compared to diabetic control.

**Table 4:** * In vivo* assessment of the antioxidant activity of *Pg, *F1, F2, and GA using catalase levels in serum of alloxan-induced diabetic mice (*n* = 7).

Group	Dose (mg/kg)	Catalase level ± SEM (kU/I)			
		1st day	3rd day	5th day	8th day
Control	—	41.00 ± 1.50	41.50 ± 1.60	40.86 ± 1.20	41.62 ± 1.70
Diabetic control^a^	—	21.67 ± 1.60***	20.93 ± 1.30***	24.01 ± 1.90***	25.55 ± 1.40***
GB^b^	5	22.60 ± 1.70	25.00 ± 1.70	31.54 ± 1.40*	32.37 ± 1.00**
*Pg* ^ b^	25	22.33 ± 1.80	22.66 ± 1.60	24.26 ± 2.10*	26.60 ± 1.90**
*Pg* ^ b^	50	20.44 ± 1.30	22.55 ± 1.90	26.22 ± 1.69*	27.10 ± 1.20**
*Pg* ^ b^	100	23.33 ± 1.50	26.40 ± 2.10	27.63 ± 2.10*	29.50 ± 2.30*
Placebo F1	200	21.50 ± 2.80	20.77 ± 2.10	20.84 ± 2.80	20.33 ± 2.20
F1^b^	50	20.11 ± 1.20	21.40 ± 1.60	24.66 ± 2.40*	26.88 ± 3.10*
F1^b^	100	22.33 ± 1.70	23.42 ± 2.20	26.10 ± 2.70*	27.10 ± 2.90*
F1^b^	200	19.44 ± 2.10	23.66 ± 2.70	27.50 ± 2.50*	30.62 ± 1.60*
Placebo F2	200	22.54 ± 2.40	20.89 ± 2.80	20.89 ± 3.10	20.66 ± 2.80
F2^b^	50	24.44 ± 2.70	24.21± 2.00	25.40 ± 1.70*	26.40 ± 1.70
F2^b^	100	20.14 ± 1.30	19.40 ± 1.40	24.00 ± 2.10*	26.56 ± 2.20*
F2^b^	200	21.85 ± 3.10	24.54 ± 2.40	26.66 ± 3.40*	28.34 ± 2.40*
GA^b^	3	21.44 ± 1.60	20.11 ± 1.10	22.36 ± 1.60	23.22 ± 1.30
GA^b^	6	20.55 ± 1.50	22.78 ± 1.60	24.85 ± 1.80*	26.42 ± 0.80*
GA^b^	12	24.00 ± 1.80	22.30 ± 1.40	24.11 ± 1.10*	26.33 ± 1.20*

SEM: standard error of the mean.

*P < 0.05 significant from the control animals.

**P < 0.01 significant from the control animals.

***P < 0.001 significant from the control animals.

^a^Compared to vehicle control.

^b^Compared to diabetic control.

## Abbreviations

DM: * Diabetes mellitus*
DN: Diabetic neuropathy
GA: Gallic acid
GB: Glibenclamide
IP: Intraperitoneal
FCR: Folin–Ciocalteu reagent
RP: Reversed phase
DMSO: Dimethyl sulfoxide
LD50: Lethality towards 50% of a population
ca.: Approximately
*P*.* granatum* (*Pg*): * Punica granatum*
ROS: Reactive oxygen species
CAT: Catalase.
